# Sex-specific associations between hemoglobin levels and incident hypertension among physically fit young adults

**DOI:** 10.3389/fcvm.2026.1902865

**Published:** 2026-09-23

**Authors:** Kun-Zhe Tsai, Ming-Wei Liu, Yi-Chiung Hsu, Wei-Nung Liu, Chuan-Tsai Tsai, Wei-Chun Huang, Gen-Min Lin

**Affiliations:** 1 Department of Medicine, Hualien Armed Forces General Hospital, Hualien City, Taiwan; 2 Department of Stomatology of Periodontology, Mackay Memorial Hospital, Taipei, Taiwan; 3 Department of Biomedical Sciences and Engineering, Center for Astronautical Physics and Engineering, National Central University, Taoyuan City, Taiwan; 4 Department of Medical Research, Cathay General Hospital, Taipei, Taiwan; 5 Department of Medicine, Tri-Service General Hospital, National Defense Medical University, Taipei, Taiwan; 6 College of Medicine, National Yang Ming Chiao Tung University, Taipei, Taiwan; 7 Cardiovascular Center, Taipei Veterans General Hospital, Taipei, Taiwan; 8 Department of Medicine, Pingtung Christian Hospital, Pingtung County, Taiwan; 9 Department of Bussiness Management, National Sun Yat-sen University, Kaohsiung, Taiwan

**Keywords:** cohort study, hemoglobin, hypertension, mild anemia, military young adults

## Abstract

**Background:**

Hemoglobin (Hb) levels have uncertain associations with blood pressure (BP). In addition, no cohort studies to date have investigated the association between Hb and hypertension among physically fit young adults.

**Methods:**

This cohort study involved 2,440 military male and female personnel, aged 18–39 years, free of baseline hypertension in Taiwan in 2014. The individuals were followed up for incident hypertension from baseline to 2020. All participants received military health examinations annually, including BP measurements. Hypertension was defined as a mean systolic BP ≥130 mmHg and/or a mean diastolic BP ≥80 mmHg, in accordance with the latest guidelines. Mild anemia was defined as Hb concentrations of 10.0–13.9 g/dL in men and 10.0–11.9 g/dL in women. Multivariable Cox regression analyses were performed, adjusting for age, sex, BP, body mass index, substance use status, serum uric acid, blood urea nitrogen, estimated glomerular filtration rate, physical activity and fitness to determine the relationships.

**Results:**

A total of 915 participants (37.5%) developed new-onset hypertension during the follow-up period. Of the 2,440 participants, 2,148 were men and 292 were women, and new-onset hypertension occurred in 871 men and 44 women. In men, Hb levels (per 1.0 g/dL increase) were positively associated with incident hypertension [hazard ratio (HR) and 95% confidence interval (CI): 1.105 (1.026–1.190)], and mild anemia was linked to a lower risk of incident hypertension [HR: 0.765 (0.597–0.979)]. The association of Hb in men were only significant for isolated diastolic hypertension [HRs: 1.176 (1.047–1.320) and 0.514 (0.325–0.813), respectively]. In young women, the associations of Hb levels and mild anemia with incident hypertension were not statistically significant (HRs: 0.765 and 1.713, both *p* > 0.05).

**Conclusions:**

Hb levels were positively associated with increased risk of hypertension among physically fit young male adults. Such association was specifically for isolated diastolic hypertension. However, among women, neither association reached significance, possibly due to the small number of hypertension events making these results uncertain.

## Introduction

Cardiovascular disease (CVD) persists as a formidable global health challenge, standing as the leading cause of mortality worldwide (1, 2). High blood pressure (BP), or hypertension, is recognized as one of the most crucial contributors to this dire situation, with recent statistics indicating that an increased proportion of CVD events were attributed to hypertension (3, 4). The nature of hypertension arises from its multifactorial etiology, encompassing genetic predispositions, obesity, diabetes mellitus, dyslipidemia, active smoking, psychosocial stressors, sedentary behavior, socioeconomic factors, and the intricate interplay between these various elements (5).

Anemia, a pathological condition characterized by diminished hemoglobin (Hb) concentration in blood circulation, frequently coexists with chronic disease and compromises the erythrocytes' delivery capacity of adequate oxygen supply to meet the metabolic requirements of tissues and organs throughout the human body (6, 7). A growing body of clinical evidence has firmly demonstrated a robust correlation between the manifestation of anemia and an increased risk of severe cardiovascular complications (8). Anemic individuals might exhibit dysregulated BP patterns, characterized by elevated nocturnal systolic BP and mean BP (9). A longitudinal follow-up study elucidated a U-shaped relationship between Hb levels and systolic BP variability, whereas a positive linear association between Hb levels and diastolic BP variability (10). As is known, most prior study cohorts included middle- or old-aged individuals in whom hypertension and anemia shared many common risk factors such as metabolic comorbidities (11, 12). Among physically fit individuals, endurance training expands plasma volume and can produce a mild dilutional decrement in Hb (“sports anemia”) (13), while lowering BP through improved endothelial function and reduced sympathetic tone (14–16). In this situation, cardiorespiratory fitness (CRF) is a vital confounder between Hb levels and hypertension, whereas rare studies adjusted baseline CRF for the association. In addition, since BP is strongly sex-dependent—premenopausal women have lower BP levels than age-matched men, partly through estrogen-mediated vasodilation—and because young women often have lower Hb levels from recurrent menstrual iron loss, sex is an important modifier which is rarely explored separately in the association between Hb levels and hypertension. In this study, we investigated the associations of Hb levels and mild anemia with incident hypertension which were also stratified by sex in a military cohort of physically fit young adults.

## Methods

### Population and setting

The occupational cohort study “Cardiorespiratory Fitness and Health in Eastern Armed Forces” (CHIEF) was conducted at the Hualien Armed Forces General Hospital, the sole referral medical institution serving eastern Taiwan. This study involved 4,080 military personnel aged 18–50 years who were recruited in 2014. The study participants exhibited a physically fit profile, being free from diabetes mellitus and not taking any antihypertensive or lipid-lowering medications at the time of enrollment into the study (17, 18). Because it formed part of the compulsory annual assessment, attendance at the bases was high. The cohort database does not record how many personnel declined, so an exact response rate for all those contacted could not be calculated. All participants underwent a thorough health examination evaluating various anthropometric measurements, hemodynamic parameters, and blood cell and biochemical profiles. In addition, via a self-report questionnaire, participants disclosed details about substance use, separating themselves into groups of active alcohol drinkers, betel-nut chewers and cigarette smokers vs. former and never-consumers (17, 19). The CHIEF study received ethical approval from the Institutional Review Board of the Mennonite Christian Hospital in Hualien, Taiwan (No. 16-05-008). All study participants provided written informed consent in accordance with established ethical standards. This research study adhered strictly to the ethical principles outlined in the Declaration of Helsinki, ensuring compliance with all relevant regulations and legal requirements throughout the entirety of the research process.

### Whole body health examination

To obtain anthropometric data, all participants were instructed to remove their footwear and required to wear lightweight clothing. Following standardized protocols with calibrated instruments, each participant's waist circumference, body mass, and height were respectively measured to the nearest 0.1 cm and 0.1 kg while the subjects assumed a standing position. Body mass index (BMI) values were then calculated by taking the ratio of an individual's body mass in kilograms to the square of their height in meters.

To assess detailed blood biochemistry, participants underwent an overnight fast exceeding 8 h. Venous blood samples were then collected and the serum component separated through centrifugation. This serum underwent precise analysis utilizing an automated Olympus AU640 analyzer. Following meticulous instructions from the kit manufacturers, the technicians measured serum concentrations of fasting glucose, triglycerides, total cholesterol, low-density and high-density lipoprotein cholesterol (LDL-C and HDL-C), uric acid, blood urea nitrogen (BUN) and creatinine (20). From the serum creatinine measurements, estimated glomerular filtration rates (eGFR) were calculated using the latest 2021 Chronic Kidney Disease Epidemiology Collaboration equation to evaluate kidney function (21).

Hematological panels including serum Hb levels, hematocrit (HCT), and mean corpuscular volume (MCV) were measured utilizing an automated hematology analyzer (Sysmex XT2000-I, Sysmex Corporation, Kobe, Japan) (22, 23).

### Definition of mild anemia

For this study on physically fit young adults, mild anemia was classified using relatively higher Hb references of 10.0–13.9 g/dL for men and 10.0–11.9 g/dL for women (6, 24, 25). These limits are relatively higher than the conventional World Health Organization anemia thresholds and were chosen to identify the mild, often dilutional fall in Hb seen in physically active young adults rather than overt clinical anemia, in keeping with earlier work in this cohort. Participants were considered non-anemic if their Hb measured 14.0 g/dL or higher in men and 12.0 g/dL or higher in women.

### Definition of hypertension and its phenotypes

The BP readings were obtained with participants in a seated position using an oscillometric automatic device from the Parama-Tech Co., Ltd (Japan). If initial systolic/diastolic BP levels exceeded 130/80 mmHg, a second measurement was taken following a 15-minute rest, with the final reported value being the average of the two readings. A second reading was thus obtained only when the first reading reached the diagnostic range, where repeat measurement and averaging do most to limit measurement error; the database retains the final value rather than the number of readings taken, so the exact one- vs. two-reading split is not available. Adhering to the American College of Cardiology and American Heart Association's guidelines (26), participants were classified as hypertensive if their averaged systolic BP was measured ≥130 mmHg and/or their diastolic BP was ≥80 mmHg and/or antihypertensive medications use. Specific hypertension phenotypes were made for isolated systolic hypertension (ISH, systolic BP ≥130 mmHg and diastolic BP <80 mmHg), isolated diastolic hypertension (IDH, systolic BP <130 mmHg and diastolic BP ≥80 mmHg), and combined hypertension (CH, systolic BP ≥130 mmHg and diastolic BP ≥80 mmHg). The cut-off levels for hypertension, stage 2 were set as the average of systolic BP of 140 mmHg and diastolic BP of 90 mmHg.

### Physical activity and CRF

Participants self-reported their moderate-intensity physical activity levels via a questionnaire focused on weekly leisure-time running duration over the prior 6 months. Responses were classified into three tiers: under 150 min/week, between 150 and 299 min/week, and 300 min/week or more.

To evaluate CRF, participants underwent an outdoor 3,000-meter non-weight bearing run test. This assessment was only conducted at 4:00 p.m. when the heat stroke risk coefficient, calculated as the product of outdoor temperature (°C) and relative humidity (%) multiplied by 0.1, was below 40 and no precipitation occurred. Each participant wore an induction bracelet, with infrared sensors at the start and end points recording running times (27–29).

### Statistical analysis

Continuous variables are presented as mean ± standard deviation, with one-way ANOVA tests comparing groups differences. Categorical data is expressed as numbers and percentages, using chi-square tests for groups comparisons.

The primary analysis utilized Cox proportional hazards regression models to evaluate the associations of mild anemia and serum Hb [treated as a continuous variable (each 1.0 g/dL increase) or as a categorical variable by quartiles] with incident hypertension by calculating hazard ratios (HRs) and 95% confidence intervals (CIs), which were stratified by sex in advance. Model 1 was the crude model. Model 2 adjusted for baseline age, sex, systolic BP, diastolic BP, BMI, substance use, uric acid, BUN and eGFR. Model 3 further adjusted for physical activity levels. Model 4 additionally adjusted for CRF levels. The associations of mild anemia and serum Hb with incident hypertension defined by stage 2 or above were treated as a sensitivity analysis. In a secondary analysis assessing anemia and Hb associations with incident ISH, IDH, and CH, adjustments were made for the covariates in Model 4. Multiple linear regression, stratified by sex and adjusted for the same covariates as Model 4, was used to relate serum hemoglobin to systolic BP and diastolic BP. Data analysis was carried out using SPSS v26.0 for Windows, the statistical software developed by IBM Corp. based in Armonk, NY. Statistical significance was defined as a *p*-value <0.05.

## Results

In 2014, the CHIEF study initially recruited 4,080 participants and commenced a 6-year longitudinal follow-up period. Of the participants, 1,024 individuals with prevalent hypertension, 58 individuals aged ≥40 years, and 8 individuals with moderate or severe anemia (Hb < 10 g/dL) were excluded. Additionally, 550 individuals who were moved out of Eastern Taiwan military bases and lost to follow-up were excluded. Consequently, the final analysis included 2,440 participants, of whom 255 (10.5%) showed mild anemia at baseline. It is notable that the baseline characteristics between the final cohort for analysis and those who lost follow-up were similar (Supplementary Table S1), which may reduce the existence of selection bias. During the study period from January 1, 2014, to December 31, 2020, we identified 915 new-onset hypertension cases (≥stage 1) and 125 hypertension cases (≥stage 2), respectively constituting 37.5% and 5.1% of the analytical cohort.

The baseline characteristics of the 2,440 participants are presented by sex in Table 1 and by baseline anemia status in Supplementary Table S2. Compared with women, men had higher Hb, HCT, systolic BP and diastolic BP, uric acid, and BMI, along with shorter run times that indicate higher CRF, whereas women had higher HDL-C and eGFR; incident hypertension (≥stage 1) was more frequent in men than in women (40.5% vs. 15.0%). Participants with baseline mild anemia had lower levels of Hb, HCT, MCV, total cholesterol, LDL-C, triglycerides, serum uric acid, and eGFR. They also exhibited a lower systolic BP, diastolic BP, BMI, and waist circumference. Additionally, these individuals had a lower proportion of active alcohol intake and cigarette smoking and had lower CRF levels.

**Table 1:** Baseline clinical characteristics of male and female participants without hypertension (*N* = 2,440).

Variables	Males (*N* = 2,148)	Females (*N* = 292)
Mild anemia^a^	215 (10.0)	40 (13.7)
Blood routine profile		
Hemoglobin, g/dL	15.11 ± 0.95	12.99 ± 1.00
(min–max)	(10.0–17.9)	(10.0–16.0)
RBC, 10^6^/uL	5.31 ± 0.45	4.68 ± 0.42
HCT, %	45.01 ± 2.51	36.62 ± 2.65
MCV, fL	85.04 ± 6.33	84.93 ± 6.77
<80 fL	202 (9.4)	41 (14.0)
80–100 fL	1,942 (90.5)	252 (86.0)
>100 fL	3 (0.1)	0 (0.0)
Blood biochemical profile		
Total cholesterol, mg/dL	172.68 ± 33.43	166.98 ± 29.73
LDL-C, mg/dL	105.04 ± 29.29	93.13 ± 25.32
HDL-C, mg/dL	47.96 ± 9.74	57.09 ± 10.92
Triglyceride, mg/dL	106.17 ± 72.33	75.87 ± 37.86
Fasting glucose, mg/dL	93.16 ± 13.23	89.07 ± 7.88
Uric acid, mg/dL	6.66 ± 1.25	4.71 ± 0.88
Blood urea nitrogen, mg/dL	12.88 ± 2.82	10.64 ± 2.41
eGFR, mL/min/1.73 m^2^	100.21 ± 13.97	110.24 ± 18.24
Age, years	28.34 ± 5.58	26.74 ± 6.00
Systolic blood pressure, mmHg	113.04 ± 9.83	105.16 ± 11.07
Diastolic blood pressure, mmHg	66.85 ± 6.97	63.89 ± 7.36
Body mass index, kg/m^2^	24.51 ± 3.03	22.49 ± 2.88
Waist circumferences, cm	82.58 ± 7.78	73.31 ± 7.44
Active substance use, %		
Alcohol drinking	909 (42.3)	52 (17.7)
Betel-nut chewing	235 (11.1)	2 (0.7)
Cigarette smoking	832 (39.1)	35 (11.9)
Physical activity, %		
<150 min/week	456 (21.2)	94 (32.1)
150–299 min/week	808 (37.6)	123 (42.0)
≥300 min/week	884 (41.2)	76 (25.9)
Cardiorespiratory fitness, seconds	855.02 ± 68.48	1,006.51 ± 76.49
Hypertension events, ≥ stage 1, %	871 (40.5)	44 (15.0)
Hypertension events, ≥ stage 2, %	115 (5.4)	10 (3.4)

Continuous variables are expressed as mean ± SD (standard deviation), and categorical variables as *N* (%). RBC, red blood cell; HCT, hematocrit; MCV, mean corpuscular volume; eGFR, estimated glomerular filtration rate; HDL-C, high -density lipoprotein; LDL-C, low-density lipoprotein cholesterol.

a Mild anemia was defined as hemoglobin of 10.0–13.9 g/dL in men and 10.0–11.9 g/dL in women.

Table 2 presents the multiple linear regression of serum Hb against BP. In men, each 1.0 g/dL higher Hb was independently associated with higher systolic BP (standardized *β* 0.047, *p* = 0.03) and higher diastolic BP (*β* 0.113, *p* < 0.001). In women, Hb was associated with diastolic BP (*β* 0.160, *p* = 0.02) but not systolic BP (*β* 0.053, *p* = 0.45). The diastolic BP association in both sexes parallels the phenotype-specific Cox results described below.

**Table 2:** Multiple linear regression analysis for the correlations of serum hemoglobin with systolic blood pressure and diastolic blood pressure.

Variables	Systolic blood pressure			Diastolic blood pressure		
	*β* (95% CI)	*R* ^2^	*p*-value	*β* (95% CI)	*R* ^2^	*p*-value
Hemoglobin (each 1.0 g/dL higher)						
Males	0.047 (0.034 to 0.938)	0.048	0.03	0.113 (0.509–1.141)	0.074	<0.001
Females	0.053 (−1.036 to 2.303)	0.087	0.45	0.160 (0.183–2.375)	0.108	0.02

Data are presented as standardized *β* and 95% confidence intervals (CI) and *R*^2^ using multiple linear regression analysis. Multivariable model: adjusted for baseline age, body mass index, substance use, uric acid, blood urea nitrogen, estimated glomerular filtration, physical activity and cardiorespiratory fitness.

Table 3 presents the results from a multivariable Cox regression analysis, which examined the association between mild anemia, Hb levels, and the incidence of new-onset hypertension (≥stage 1) during a median follow-up period of 6 years. After full adjustments including CRF levels (Model 4), those with mild anemia retained a significantly lower risk of incident hypertension compared with non-anemic participants [HR: 0.765 (95% CI: 0.597–0.979), *p* = 0.03]. Correspondingly, each 1.0 g/dL increment in baseline Hb was independently associated with a 10.0% greater risk of incident hypertension [HR: 1.100 (95% CI: 1.023–1.183), *p* = 0.01].

**Table 3:** Multivariable Cox regression analysis for incident hypertension (≥ stage 1) with baseline mild anemia and serum hemoglobin.

Variables	Model 1		Model 2		Model 3		Model 4	
	HR (95% CI)	*p*-value	HR (95% CI)	*p*-value	HR (95% CI)	*p*-value	HR (95% CI)	*p*-value
Hemoglobin (each 1.0 g/dL higher)	1.272 (1.192–1.356)	<0.001	1.102 (1.024–1.184)	0.009	1.100 (1.022- 1.183)	0.01	1.100 (1.023–1.183)	0.01
Males	1.158 (1.074–1.249)	<0.001	1.106 (1.027–1.191)	0.008	1.104 (1.025–1.189)	0.009	1.105 (1.026–1.190)	0.008
Females	1.037 (0.704–1.527)	0.85	0.768 (0.499–1.181)	0.22	0.763 (0.491–1.186)	0.22	0.765 (0.491–1.192)	0.23
Mild anemia^a^	0.742 (0.582–0.947)	0.01	0.766 (0.599–0.981)	0.03	0.770 (0.601–0.985)	0.03	0.765 (0.597–0.979)	0.03
Males	0.750 (0.583–0.963)	0.02	0.752 (0.584–0.970)	0.02	0.756 (0.586–0.974)	0.03	0.751 (0.582–0.968)	0.02
Females	0.966 (0.337–2.771)	0.94	1.690 (0.525–5.438)	0.37	1.735 (0.527–5.704)	0.36	1.713 (0.514–5.710)	0.38

Data are presented as hazard ratios (HR) and 95% confidence intervals (CI) using multivariable Cox regression analysis. Model 1: crude model. Model 2: adjusted for baseline age, sex, systolic blood pressure, baseline diastolic blood pressure, body mass index, substance use, uric acid, blood urea nitrogen and estimated glomerular filtration. Model 3: adjusted for variables in the Model 2 and physical activity. Model 4: adjusted for variables in the Model 3 and cardiorespiratory fitness.

a Mild anemia was defined as hemoglobin of 10.0–13.9 g/dL in men and 10.0–11.9 g/dL in women.

If serum Hb was treated as a categorical variable, the results for the incidence of hypertension (≥stage 1) increased with higher Hb quartiles (*p*-value for trend: 0.001 in crude model and 0.07 in other models), which were in line with the results using Hb treated as a continuous variable (Supplementary Table S3). On the contrary, in sensitivity analysis, there were no associations of mild anemia and serum Hb (treated as a continuous variable) with incident hypertension (≥stage 2). There was a U-shaped relationship between serum Hb (classified by quartiles) and incident hypertension (≥stage 2) in Supplementary Table S3.

Table 3 also shows the sex-specific association between mild anemia, Hb levels, and incident hypertension (≥stage 1). In men, mild anemia was associated with a lower risk of incident hypertension (HR 0.751 [95% CI 0.582–0.968], *p* = 0.02) and Hb levels (each 1.0 g/dL increase) were positively associated with incident hypertension [HR: 1.105 (95% CI 1.026–1.190), *p* = 0.008]. On the contrary, both associations in women ran in the opposite direction to men but did not reach statistical significance: each 1.0 g/dL higher Hb level was associated with a numerically lower hypertension risk [HR 0.765 (95% CI 0.491–1.192), *p* = 0.23], whereas mild anemia was associated with a numerically higher risk [HR 1.713 (95% CI 0.514–5.710), *p* = 0.38]. The *p*-values for interaction within the sex were not significant for either Hb levels (*p* = 0.82) or mild anemia (*p* = 0.65), which might be due to the wide CIs in women.

Table 4 shows the multivariable-adjusted associations of mild anemia and Hb levels with the risk of different hypertension phenotypes (≥stage 1) in men. Mild anemia and Hb levels demonstrated a pattern of hypertension phenotypic specificity: associations were statistically significant for IDH while not for ISH or CH. Mild anemia was linked to a lower risk of IDH [HR: 0.514 (95% CI: 0.325–0.813), *p* = 0.004], while higher serum Hb levels (each 1.0 g/dL increase) were linked to a higher risk of IDH [HR: 1.176 (95% CI: 1.047–1.320), *p* = 0.006]. Phenotype-specific models could not be estimated in women: the 44 incident hypertension events among female participants were too few to support stable multivariable estimation once divided across the three phenotypes, so Table 4 is restricted to men.

**Table 4:** Associations of mild anemia and serum hemoglobin With incident hypertension phenotypes (≥ stage 1) in men.

Variables	Isolated systolic hypertension (*N* = 322)		Isolated diastolic hypertension (*N* = 358)		Combined hypertension (*N* = 191)	
	HR (95% CI)	*p*-value	HR (95% CI)	*p*-value	HR (95% CI)	*p*-value
Hemoglobin (each 1.0 g/dL higher)	1.041 (0.917–1.182)	0.53	1.176 (1.047–1.320)	0.006	1.084 (0.921–1.275)	0.33
Mild anemia^a^	0.823 (0.539–1.256)	0.36	0.514 (0.325–0.813)	0.004	1.083 (0.683–1.716)	0.73

Data are presented as hazard ratios (HR) and 95% confidence intervals (CI) using multivariable Cox regression analysis adjusting for baseline age, sex, systolic blood pressure, baseline diastolic blood pressure, body mass index, substance use, uric acid, blood urea nitrogen and estimated glomerular filtration.

a Mild anemia was defined as hemoglobin of 10.0–13.9 g/dL in men and 10.0–11.9 g/dL in women.

## Discussion

In this cohort study of 2,440 physically fit young adults, there were three main findings. First, Hb levels were positively associated with incident hypertension (≥stage 1) in men with adjustment for multiple covariates including objectively measured CRF, a covariate rarely controlled in prior studies, and the positive findings among men were only confined to the hypertension subtype of IDH. Second, there is a U-shaped relationship between serum Hb and the incidence of hypertension once the diagnostic threshold was raised to 140/90 mmHg (≥stage 2) among these young adults.

For the correlation between Hb and BP levels, a Dutch national study reported a positive correlation in both healthy men and women (30). Several other studies have also shown similar results (31–33). This relationship may be mediated by BMI and waist circumference (34). Animal studies have revealed that mean arterial BP rises with higher infusion rates of cross-linked Hb (35). Another large-scale cross-sectional study reported bidirectional causal relationships between Hb and red blood cells with diastolic BP, but not with systolic BP, using bidirectional two-sample Mendelian randomization analyses (36), which were in line with our study findings that Hb levels were associated with a higher risk of IDH while not ISH in physically fit males. One study found that hypertensive elderly individuals had elevated nocturnal systolic BP and mean BP and a trend for higher diastolic BP (12). Furthermore, a prior 7-year follow-up study suggests that Hb *per se* does not cause the development of hypertension in their no hypertensive subcohort at baseline on longitudinal analysis, although the mean Hb was significantly higher in those with hypertension than in those without hypertension on cross-sectional analysis (37). That study, however, was conducted in a rural community of Korean adults aged 35–88 years, analyzed hypertension as a single outcome, and did not account for CRF. The difference from our longitudinal data likely reflects these distinctions: our participants were younger and uniformly fit, we measured and adjusted for CRF, and our positive association was limited to the definition of hypertension using the broader range of BP (≥stage 1) and present in only IDH subtype. Notwithstanding, a longitudinal relationship between Hb levels and incident hypertension has not been fully elucidated, which requires further investigation.

Hb is intricately associated with BP regulation through its influence on arterial stiffness and nitric oxide (NO) bioavailability (30). Elevated Hb concentrations may increase arterial stiffness, a condition that augments both systolic BP and diastolic BP by reducing arterial compliance and escalating resistance to blood flow (38). Free Hb in the circulation acts as a scavenger of NO, a crucial molecule synthesized by endothelial cells lining the blood vessels. NO plays a pivotal role in inducing relaxation of the smooth muscle cells surrounding the vessels, thereby facilitating vasodilation and modulating BP. When free Hb binds to NO, it reduces the bioavailability of NO, leading to vasoconstriction and a subsequent elevation in BP (39). This pathway may explain the phenotype-specific finding in our study: because diastolic BP is more dependent on peripheral vascular resistance than systolic BP, reduced NO bioavailability would tend to manifest as IDH.

The protective effect of mild anemia on incident hypertension diverges from the traditional view of anemia as unfavorable. Two explanations may account for this finding, although neither could be confirmed here because iron-status markers were not measured. First, athlete's pseudo-anemia (or sports anemia) is well documented: habitual endurance training induces plasma volume expansion of 10%–20% (13), diluting circulating Hb without reducing total red cell mass or oxygen-carrying capacity. Such dilutional anemia reflects training status rather than a deficiency state (6), and its link to lower BP likely reflects training adaptations such as improved endothelial function, reduced sympathetic hyperactivity, and increased arterial compliance (14–16), rather than a direct effect of lowered Hb. Second, at the lower end of the physiologic Hb range, blood viscosity falls and NO bioavailability rises, reducing vascular resistance independently of training status (38, 39).

The relationship between serum Hb and hypertension also changed once we tightened the BP cut-off. At the conventional 140/90 mmHg level (≥stage 2), Hb no longer predicted incident hypertension when entered as a continuous term, and the quartile analysis instead showed a U-shaped pattern, with the lowest and the highest Hb quartiles both carrying more risk than the middle quartiles (Supplementary Table S4). This fits what is known about the two ends of the Hb range. High serum Hb raises vascular resistance through greater blood viscosity and NO scavenging, as discussed above, while anemia tends to raise cardiac output and often accompanies other conditions that push BP up. A similar U-shape with incident hypertension was recently reported for the white blood cell count in this cohort (40), and we have previously described a U-shaped link between Hb and long-term systolic BP variability in these men (10). Part of the inconsistency in earlier reports, many of which used the 140/90 definition and entered Hb as a linear term (37), may simply reflect this curvature, which a single linear term cannot pick up. The results for stage 2 hypertension or greater should still be read cautiously, since only 125 participants reached this threshold and the estimates at the extremes of the Hb range were imprecise. Measuring iron-status markers would help, by separating dilutional from iron-deficient anemia at the low end, while rerunning the analysis under both the 130/80 and 140/90 mmHg definitions of hypertension would make the findings easier to set against older cohorts.

The interpretation for the uncertain association between Hb levels and incident hypertension in young women should be cautious. Since only 40 women were found to have mild anemia and hypertension events among women were few, the CIs were wide and neither the sex-by-Hb nor the sex-by-anemia interaction reached significance. The BP in young women is strongly governed by ovarian hormones: estrogen stimulates endothelial synthesis of NO which can promote vasodilation, and thus premenopausal women have lower vascular resistance and BP than age-matched men (41, 42). This vasodilatory background, along with the lower NO-scavenging burden that comes with women's lower circulating Hb, may outweigh the modest pressor effect of Hb on peripheral resistance and so weaken the Hb–BP relationship in young women. Also, a lower Hb more often reflects recurrent menstrual iron loss and iron-deficient erythropoiesis (43) in menstruating women whose hemodynamics differ from those of dilutional pseudo-anemia in physically fit men. These interpretations remain tentative; confirming them will require sufficient powered, sex-specific cohorts that also record menstrual status, hormonal contraception, and iron-status biomarkers.

The main strengths of this study were that it represents one of a few studies to adjust baseline physical activity and CRF levels when assessing the association between Hb levels and incident hypertension. Nevertheless, several limitations of this study must be acknowledged. First, because the observational nature of this research, a causal effect between Hb levels and the development of new-onset hypertension cannot be firmly established. Second, the study population included young, physically fit military personnel, predominantly male (88%), with lower BMI and higher CRF levels than the general Taiwanese population of comparable age and sex (44). Although this homogeneity strengthens internal validity by reducing confounding from physical deconditioning, it restricts generalizability to sedentary individuals, women, and the elderly. Third, no information was available for family history, dietary sodium, sleep quality and disorders, psychosocial stress, inflammatory status, and the potential presence of secondary hypertension, which may be unrelated to mild anemia, thereby introducing potential bias. Fourth, iron-status markers such as ferritin, serum iron, transferrin saturation, and reticulocyte counts were not measured, so we cannot separate dilutional pseudo-anemia from iron-deficiency or other causes related anemia, rendering the mechanistic inference speculative in young men. Fifth, a non-standard BP measurement protocol with selective repeat readings only for participants with elevated BP values, whose potential bias cannot be quantified since the number of readings per participant is not recorded. Finally, the statistical power for phenotype-specific analyses in women and for stage 2 hypertension analyses was insufficient, as reflected by imprecise estimates at the extremes of the hemoglobin distribution.

## Conclusions

Serum Hb levels were positively associated with increased risk of hypertension (≥stage 1) in physically fit young men. Such association was specifically for IDH. However, in young women, neither association reached significance, possibly due to the small number of hypertension events making these results uncertain. A larger study including more young women would be required to clarify the association between Hb levels and incident hypertension. In clinical practice, routine assessment of Hb may add value to early stratification of hypertension risk among physically fit young adults, in whom conventional risk calculators perform poorly as these risk factors are largely absent at young ages. Whether the protective association of mild anemia in men is related to training-related physiology requires further investigation.

## Data Availability

The raw data supporting the conclusions of this article will be made available by the authors, without undue reservation.
