# Supplementary material for: Sex-specific associations between hemoglobin levels and incident hypertension among physically fit young adults
Source: Front Cardiovasc Med. 2026 Sep 23;13:1902865. doi: 10.3389/fcvm.2026.1902865 (PMC13642576; doi:10.3389/fcvm.2026.1902865)
Supplement: Supplementary file 1 [file Datasheet1.docx]

Supplemental Table 1. Baseline Clinical Characteristics of the Study Cohort and Participants Who Lost Follow-up

|  | The Study Cohort for Analysis  (N =2,440) | Participants Who Lost Follow-up  (N =550) | P-Value |
| --- | --- | --- | --- |
| *Mild anemia | 255 (10.0) | 66 (12.0) | 0.28 |
| Blood routine profile |  |  |  |
| Hemoglobin, g/dL | 14.85 ± 1.86 | 14.83 ± 1.20 | 0.71 |
| (min – max) | (8.6 – 17.9) | (8.6 – 17.6) |  |
| RBC, 10^6^/uL | 5.24 ± 0.49 | 5.22 ± 0.48 | 0.44 |
| HCT, % | 44.36 ± 3.07 | 44.27 ± 3.05 | 0.50 |
| MCV, fL | 85.03 ± 6.37 | 85.16 ± 6.67 | 0.68 |
| <80 fL | 242 (9.9) | 60 (10.9) | 0.75 |
| 80 – 100 fL | 2195 (90.0) | 489 (88.9) |  |
| >100 fL | 3 (0.1) | 1 (0.2) |  |
| Blood biochemical profile |  |  |  |
| Total cholesterol, mg/dL | 172.00 ± 33.06 | 169.89 ± 33.40 | 0.17 |
| LDL-C, mg/dL | 103.62 ± 29.10 | 102.01 ± 29.24 | 0.24 |
| HDL-C, mg/dL | 49.06 ± 10.32 | 49.10 ± 10.15 | 0.93 |
| Triglyceride, mg/dL | 102.51 ± 69.81 | 99.57 ± 58.07 | 0.21 |
| Fasting glucose, mg/dL | 92.68 ± 12.77 | 92.71 ± 15.21 | 0.96 |
| Uric acid, mg/dL | 6.43 ± 1.36 | 6.27 ± 1.28 | 0.01 |
| Blood urea nitrogen, mg/dL | 12.61 ± 2.87 | 12.73 ± 2.92 | 0.39 |
| eGFR, mL/min/1.73m^2^ | 101.41 ± 14.91 | 101.39 ± 15.28 | 0.97 |
| Age, years | 28.15 ± 5.66 | 28.64 ± 5.45 | 0.07 |
| Male sex, % | 2148 (88.0) | 497 (90.4) | 0.11 |
| Systolic blood pressure, mmHg | 112.10 ± 10.31 | 111.56 ± 10.33 | 0.27 |
| Diastolic blood pressure, mmHg | 66.50 ± 7.08 | 66.57 ± 6.86 | 0.82 |
| Body mass index, kg/m^2^ | 24.26 ± 3.08 | 24.16 ± 3.00 | 0.47 |
| Waist circumferences, cm | 81.48 ± 8.30 | 81.32 ± 8.24 | 0.69 |
| Active substance use, % |  |  |  |
| Alcohol drinking | 961 (39.4) | 203 (36.9) | 0.29 |
| Betel-nut chewing | 237 (9.7) | 46 (8.4) | 0.32 |
| Cigarette smoking | 867 (35.5) | 192 (34.9) | 0.79 |
| Physical activity, % |  |  |  |
| <150 min/week | 550 (22.5) | 115 (20.9) | 0.30 |
| 150 – 299 min/week | 931 (38.2) | 229 (41.6) |  |
| ≥300 min/week | 960 (39.3) | 206 (37.5) |  |
| Cardiorespiratory fitness, seconds | 870.73 ± 83.32 | 870.74 ± 78.52 | 0.99 |

Continuous variables are expressed as mean ± SD (standard deviation), and categorical variables as N (%)

Abbreviations: RBC, red blood cell; HCT, hematocrit; MCV, mean corpuscular volume; eGFR, estimated glomerular filtration rate; HDL-C, high -density lipoprotein; LDL-C, low-density lipoprotein cholesterol.

*Mild anemia was defined as hemoglobin of 10.0-13.9 g/dL in men and 10.0-11.9 g/dL in women

Supplemental Table 2. Baseline Clinical Characteristics of Participants Without Hypertension (N =2,440)

|  | No anemia  (N =2,185) | Mild anemia*  (N =255) |
| --- | --- | --- |
| Blood routine profile |  |  |
| Hemoglobin, g/dL | 15.08 ± 0.98 | 12.94 ± 1.01 |
| (Range: min – max) | (12.0 – 17.9) | (10.0 – 13.9) |
| RBC, 10^6^/uL | 5.23 ± 0.43 | 5.28 ± 0.86 |
| HCT, % | 44.89 ± 2.65 | 39.83 ± 2.72 |
| MCV, fL | 85.97 ± 4.74 | 79.94 ± 11.16 |
| <80 fL | 120 (5.5) | 122 (48.0) |
| 80 – 100 fL | 2062 (94.4) | 133 (52.0) |
| >100 fL | 3 (0.1) | 0 (0.0) |
| Blood biochemical profile |  |  |
| Total cholesterol, mg/dL | 173.06 ± 33.11 | 162.88 ± 31.13 |
| LDL-C, mg/dL | 104.42 ± 29.26 | 96.71 ± 26.80 |
| HDL-C, mg/dL | 49.07 ± 10.27 | 48.98 ± 10.70 |
| Triglyceride, mg/dL | 104.24 ± 68.91 | 87.95 ± 75.61 |
| Fasting glucose, mg/dL | 92.73 ± 13.06 | 92.18 ± 9.98 |
| Uric acid, mg/dL | 6.45 ± 1.34 | 6.21 ± 1.54 |
| Blood urea nitrogen, mg/dL | 12.62 ± 2.84 | 12.50 ± 3.09 |
| eGFR, mL/min/1.73m^2^ | 101.03 ± 14.45 | 104.67 ± 18.09 |
| Age, years | 28.08 ± 5.62 | 28.75 ± 5.97 |
| Male sex, % | 1933 (88.5) | 215 (84.0) |
| Systolic blood pressure, mmHg | 112.24 ± 10.26 | 110.84 ± 10.65 |
| Diastolic blood pressure, mmHg | 66.65 ± 7.03 | 65.21 ± 7.38 |
| Body mass index, kg/m^2^ | 24.31 ± 3.07 | 23.83 ± 3.12 |
| Waist circumferences, cm | 81.60 ± 8.31 | 80.42 ± 8.14 |
| Active substance use, % |  |  |
| Alcohol drinking | 877 (40.1) | 84 (32.8) |
| Betel-nut chewing | 220 (10.2) | 17 (6.6) |
| Cigarette smoking | 792 (36.6) | 75 (29.3) |
| Physical activity, % |  |  |
| <150 min/week | 494 (22.6) | 56 (21.9) |
| 150 – 299 min/week | 832 (38.1) | 99 (38.7) |
| ≥300 min/week | 859 (39.3) | 101 (39.5) |
| Cardiorespiratory fitness, seconds | 869.05 ± 82.30 | 885.09 ± 90.44 |
| Hypertension events, ≥ stage 1, % | 836 (38.3) | 79 (30.9) |
| Hypertension events, ≥ stage 2, % | 112 (5.1) | 13 (5.1) |

Continuous variables are expressed as mean ± SD (standard deviation), and categorical variables as N (%)

Abbreviations: RBC, red blood cell; HCT, hematocrit; MCV, mean corpuscular volume; eGFR, estimated glomerular filtration rate; HDL-C, high -density lipoprotein; LDL-C, low-density lipoprotein cholesterol.

*Mild anemia was defined as hemoglobin of 10.0-13.9 g/dL in men and 10.0-11.9 g/dL in women

Supplemental Table 3. Multivariable Cox Regression Analysis for Incident Hypertension (≥Stage 1) With Baseline Mild Anemia and Hemoglobin

|  | Model 1 |  |  | Model 2 |  |  | Model 3 |  |  | Model 4 |  |
| --- | --- | --- | --- | --- | --- | --- | --- | --- | --- | --- | --- |
|  | HR (95% CI) | p-value |  | HR (95% CI) | p-value |  | HR (95% CI) | p-value |  | HR (95% CI) | p-value |
| Hemoglobin Q1 | 1.000 |  |  | 1.000 |  |  | 1.000 |  |  | 1.000 |  |
| Hemoglobin Q2 | 1.075 (0.876 – 1.320) | 0.48 |  | 1.120 (0.912 – 1.376) | 0.28 |  | 1.118 (0.910 – 1.374) | 0.28 |  | 1.123 (0.914 – 1.380) | 0.26 |
| Hemoglobin Q3 | 1.204 (0.989 – 1.466) | 0.065 |  | 1.178 (0.967 – 1.435) | 0.10 |  | 1.172 (0.961 – 1.429) | 0.11 |  | 1.174 (0.963 – 1.432) | 0.11 |
| Hemoglobin Q4  P for trend | 1.388 (1.138 – 1.693) | 0.001  0.001 |  | 1.200 (0.980 – 1.469) | 0.07  0.07 |  | 1.195 (0.975 – 1.463) | 0.08  0.07 |  | 1.197 (0.978 – 1.467) | 0.08  0.07 |

Data are presented as hazard ratios (HR) and 95% confidence intervals (CI) using multivariable Cox regression analysis

*Mild anemia was defined as hemoglobin of 10.0-13.9 g/dL in men and 10.0-11.9 g/dL in women.

Stage 1 hypertension is defined as systolic blood pressure ≥130 mmHg, and/or diastolic blood pressure ≥80 mmHg, and/or the use of antihypertensive medications

Q1 (N =525/73): hemoglobin <14.6 g/dL in men; <12.4 g/dL in women

Q2 (N =528/75): hemoglobin 14.6 - 15.1 g/dL in men; 12.4 - 13.0 g/dL in women

Q3 (N =584/75): hemoglobin 15.2 - 15.7 g/dL in men; 13.1 - 13.6 g/dL in women

Q4 (N =511/70): hemoglobin ≥15.8 g/dL in men; ≥13.7 g/dL in women

Model 1: crude model

Model 2: adjusted for age, sex, baseline systolic blood pressure, baseline diastolic blood pressure, body mass index, substance use, uric acid, blood urea nitrogen and estimated glomerular filtration rate

Model 3: adjusted for variables in the Model 2 and physical activity

Model 4: adjusted for variables in the Model 3 and cardiorespiratory fitness

Supplemental Table 4. Multivariable Cox Regression Analysis for Incident Hypertension (≥Stage 2) With Baseline Mild Anemia and Hemoglobin

|  | Model 1 |  |  | Model 2 |  |  | Model 3 |  |  | Model 4 |  |
| --- | --- | --- | --- | --- | --- | --- | --- | --- | --- | --- | --- |
|  | HR (95% CI) | p-value |  | HR (95% CI) | p-value |  | HR (95% CI) | p-value |  | HR (95% CI) | p-value |
| Hemoglobin | 1.181 (0.991 – 1.407) | 0.064 |  | 1.077 (0.881 – 1.316) | 0.46 |  | 1.072 (0.878 – 1.310) | 0.49 |  | 1.073 (0.878 – 1.310) | 0.49 |
| (each 1.0 g/dL higher) |  |  |  |  |  |  |  |  |  |  |  |
| Hemoglobin Q1 | 1.000 |  |  | 1.000 |  |  | 1.000 |  |  | 1.000 |  |
| Hemoglobin Q2 | 0.760 (0.432 – 1.339) | 0.34 |  | 0.824 (0.467 – 1.453) | 0.50 |  | 0.816 (0.462 – 1.441) | 0.48 |  | 0.817 (0.463 – 1.443) | 0.48 |
| Hemoglobin Q3 | 0.845 (0.493 – 1.449) | 0.54 |  | 0.851 (0.495 – 1.463) | 0.55 |  | 0.849 (0.493 – 1.461) | 0.55 |  | 0.849 (0.493 – 1.461) | 0.55 |
| Hemoglobin Q4  P for trend | 1.203 (0.718 – 2.013) | 0.48  0.45 |  | 1.029 (0.609 – 1.739) | 0.91  0.90 |  | 1.020 (0.604 – 1.725) | 0.94  0.91 |  | 1.021 (0.604 – 1.727) | 0.93  0.91 |
| *Mild anemia | 1.024 (0.561 – 1.870) | 0.93 |  | 1.012 (0.549 – 1.862) | 0.97 |  | 1.015 (0.550 – 1.871) | 0.96 |  | 1.012 (0.548 – 1.869) | 0.96 |

Data are presented as hazard ratios (HR) and 95% confidence intervals (CI) using multivariable Cox regression analysis

*Mild anemia was defined as hemoglobin of 10.0-13.9 g/dL in men and 10.0-11.9 g/dL in women.

Stage 2 hypertension is defined as systolic blood pressure ≥130 mmHg, and/or diastolic blood pressure ≥80 mmHg, and/or the use of antihypertensive medications

Q1 (N =525/73): hemoglobin <14.6 g/dL in men; <12.4 g/dL in women

Q2 (N =528/75): hemoglobin 14.6 - 15.1 g/dL in men; 12.4 - 13.0 g/dL in women

Q3 (N =584/75): hemoglobin 15.2 - 15.7 g/dL in men; 13.1 - 13.6 g/dL in women

Q4 (N =511/70): hemoglobin ≥15.8 g/dL in men; ≥13.7 g/dL in women

Model 1: crude model

Model 2: adjusted for age, sex, baseline systolic blood pressure, baseline diastolic blood pressure, body mass index, substance use, uric acid, blood urea nitrogen and estimated glomerular filtration rate

Model 3: adjusted for variables in the Model 2 and physical activity

Model 4: adjusted for variables in the Model 3 and cardiorespiratory fitness
